# Electrochemical Formation Mechanism of Microdroplets on Pure Iron

**DOI:** 10.3389/fchem.2021.610738

**Published:** 2021-04-14

**Authors:** Xiao Tang, Juanjuan Li, Yuan Wu, Hao Hu, Chao Ran Ma, Yan Li, Haiming Fan

**Affiliations:** ^1^ Shandong Key Laboratory of Oilfield Chemistry, China University of Petroleum (East China), Qingdao, China; ^2^ School of Materials Science and Engineering, China University of Petroleum (East China), Qingdao, China; ^3^ Rizhao Shihua Crude Oil Terminal Co., Ltd., Rizhao, China

**Keywords:** microdroplets, quartz crystal microbalance, microscopic observation, atmospheric corrosion, concentric three-electrode array

## Abstract

The electrochemical formation mechanism of microdroplets formed around a primary droplet of 3.5% NaCl solution on an iron-plated film was investigated by quartz crystal microbalance (QCM) and concentric three-electrode array (CTEA) measurements. During the initial stage, the microdroplets mainly originate from evaporation owing to cathodic polarization and electric current of the localized corrosion cell under the primary droplet. The maximal electrochemical potential difference between the anode and cathode was measured to be 0.36 V and acted as the driving force for the formation of microdroplets. The maximums of anodic and cathodic electric current density of pure iron under the NaCl droplet are 764 and −152 μA/cm^2^, respectively. Propagation of microdroplets in the developing stage attributes to horizontal movement of the electrolyte, water evaporation, and recondensation from primary and capillary condensation from moist air. The results of the study suggest that the initiation and propagation of microdroplets could promote and accelerate marine atmospheric corrosion.

## Introduction

Steel structures in coastal regions experience marine atmospheric corrosion, which is affected by factors such as the presence of pollutants, temperature, relative humidity (RH), and wind. In a simulation of atmospheric corrosion on carbon steel, Wang et al. observed the formation of tiny microdroplets around a macroscopic salt solution droplet (Wang and Tsuru, 2003). Neufeld et al. (2002) reported a similar phenomenon and called it the secondary spreading effect.

A number of investigators have focused on the microdroplet formation phenomenon, which is closely related to atmospheric corrosion. Studies on the microdroplets have been conducted from various perspectives such as chemistry and growth characteristics (Tsuru et al., 2004), electrochemical investigations (Zhang et al., 2005), microscopic observations (Bian et al., 2005), the effect of electrochemical polarization on the microdroplet formation (Wang et al., 2008), and the effect of the properties of the primary droplet on the microdroplets (Liang et al., 2006). It was found that cathodic polarization can initiate and accelerate the formation of microdroplets, whereas anodic polarization can inhibit the microdroplets formation.

However, the formation mechanism of the microdroplets has not been clarified. In particular, the origin of the microdroplets is not understood. Tsuru et al. (2004) proposed that the expansion of the wet area may be attributed to water movement from the primary droplet. In contrast, Liang et al. (2006) suggested that the water vapor evaporated from the primary droplet into the air condenses onto the metal surface adjacent to the three-phase boundary to form microdroplets. Corrosion could not initiate under relatively small NaCl-electrolyte droplets with a critical size of approximately 40–100 μm for low-carbon steel, whereas 150–200 μm for ultrapure iron (Li and Hihara, 2016).

Droplets have been commonly used as a typical electrolyte system for the study of the atmospheric corrosion of iron (Risteen et al., 2014), carbon steel (Han et al., 2013; Schindelholz et al., 2014; Wang et al., 2016), stainless steel (Hastuty et al., 2010; Wang et al., 2011; Cook et al., 2017; Guo et al., 2019), zinc (Azmat et al., 2011; Li and Hihara, 2014), aluminum alloy (Yan et al., 2016; Bonzom and Oltra 2017; Thomson and Frankel, 2017), and copper (Lin and Frankel, 2013). Despite historically being the focus of much research, atmospheric corrosion under droplets and its underlying mechanisms continues to require more accurate investigations. Water drop corrosion was first reported by Evans in 1926 (Evans, 1960). According to the Evans water drop experiment, areas near the periphery of a drop have ready access to oxygen from the air and function as cathodes, whereas areas under the center of the drop have less access to oxygen and function as anodes.

As a typical nonuniform corrosion electrolyte, a saline droplet may produce a heterogeneous electrochemical distribution and affect the corrosion electrode process in the initial stage of atmospheric corrosion. Generally, under the influence of changes in atmospheric conditions like temperature and humidity, droplets are evaporated or condensed in the initial stage of atmospheric corrosion, thus existing in the form of dynamic droplets. Recently, we investigated the electrochemical response to a dynamic 3.5% NaCl droplet on pure iron (Tang et al., 2019). The results showed that dynamic droplets with increasing area coverage could promote and accelerate the initiation and propagation of atmospheric corrosion. However, details regarding the electrochemical corrosion process under the droplet and its role in microdroplet formation have not been clearly determined. On the contrary, the formation and expansion of microdroplets will inevitably affect the morphology of the primary droplet and the electrochemical process of corrosion under the droplet.

The formation of microdroplets around the electrolyte droplet could be helpful in spreading the main droplet solution (Li and Hihara, 2016). Thus, the formation of microdroplets may promote marine atmospheric corrosion. The aim of this article is to investigate the formation mechanism of microdroplets during the initial stages of marine atmospheric corrosion using the quartz crystal microbalance technique, concentric three-electrode array, and microscopic observations.

## Experimental

### Measurement of Mass Change in the Microdroplet System

The mass change of the microdroplets system, which includes the primary droplet and surrounding microdroplets, was measured by a CHI 440 QCM. The quartz electrode was a thin circular AT-cut quartz crystal plate sandwiched between gold films deposited by chemical vapor deposition. The fundamental oscillation frequency *f*_*0*_ of the quartz crystal was 10 ± 0.04 MHz, and the mass sensitivity was 0.226 Hz·cm^2^/ng. Prior to the experiments, an iron layer was electroplated onto the surface of the gold electrode at a current density of 10 mA/cm^2^ for 120 s at room temperature using an iron plating solution (1.0 M FeSO_4_ + 0.75 M (NH_4_)_2_SO_4_ solution with some special additives). The iron-plated QCM electrode was subsequently cleaned with distilled water and acetone.

The QCM electrode was placed in the air portion of a sealed container, which was filled with saturated KCl solution at the bottom so as to maintain a relative humidity of 85% at room temperature. Subsequently, a 3.5% NaCl droplet with a volume of 1.0 μL or 4.0 μL was dropped onto the center of the iron film, respectively.

### Measurement of Local Electrochemical Distribution

**FIGURE 1:**
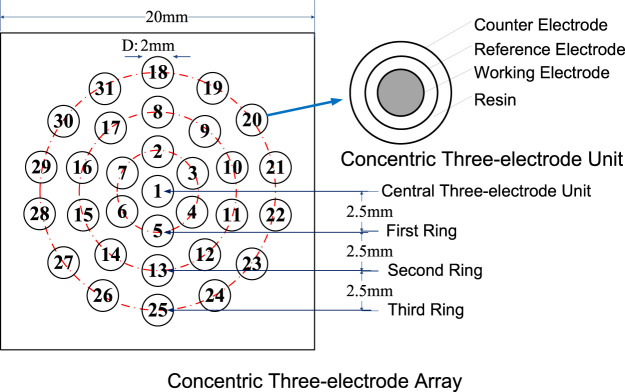
Schematic representation of the CTEA.

A schematic representation of the newly developed CTEA is shown in Figure 1. The center of the individual CTEA unit was a pure iron wire with a diameter of 0.5 mm, used as the working electrode. A ring-shaped pure zinc foil and a Pt foil were used as the reference and counter electrodes, respectively. Pure zinc had a stable potential of −0.763 V vs. SHE in 3.5% NaCl solution. The three electrodes were separated by an insulating material. Thirty-one electrode units with outer diameter of 2 mm were arranged in a concentric circular array.

Galvanic current and corrosion potential were tested using a home-built local electrochemical test system consisting of a PXI controller, high-speed transfer switches, and digital multimeters from National Instruments.

The corrosion potential distribution test was performed as per the following procedure. First, all the working electrodes of the CTEA were coupled. A three-electrode unit was extracted sequentially, the other electrode units were maintained in coupled state, and the potential difference between the working and reference electrodes was measured. Then, the corrosion potentials of the other units were measured, and, finally, the corrosion potentials of the CTEA were obtained. Pure zinc had a stable potential of −763 ± 5 mV vs. SHE under a 3.5% NaCl droplet. Under a NaCl droplet in an open environment, corrosion potentials of Zn foil references at different locations are relatively similar, and the maximum potential difference is less than 10 mV. This result shows that factors such as local solution chemistry and electrolyte thickness have little effect on the corrosion kinetics of zinc.

When measuring the galvanic current, each electrode unit was sequentially extracted and the other electrode units were coupled. Then, the galvanic current between the extracted unit and the other coupled units was measured, and the other units were sequentially measured to obtain galvanic current distribution.

### Microscopic and Macroscopic Observation of the Microdroplets

Microscopic imagines of the NaCl droplets dropped onto the iron-plated QCM electrode surface were observed with a confocal laser scanning microscope (LEXT OLS4100). Furthermore, a stereomicroscope was used to carry out morphological observation of microdroplets system at different time after dropping the primary droplet.

### Chemical Composition Measurement of the Microdroplets

The pure iron specimens used for chemical composition examination were subjected to a 3.5% NaCl droplet for different elapsed times (1, 5, 10, 20, and 45 min). Then, they were dried at a temperature of 333 K. The analysis was performed on a JEOM JSM-7610F scanning electron microscope with an energy X-ray spectrometer. The accelerating voltage was 15 kV.

## Results

### Microscopic Observations

**FIGURE 2:**
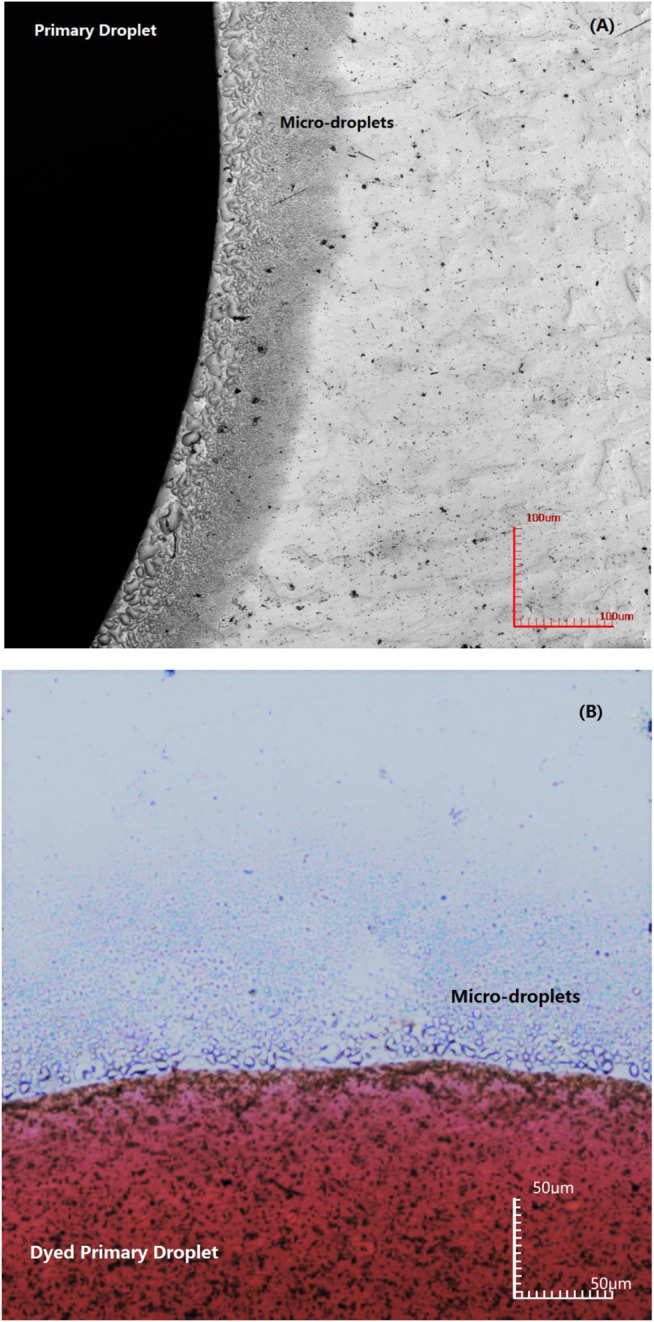
Image of initial microdroplets formed on an iron-plated layer by confocal laser scanning microscope for different primary droplets: **(A)** 3.5% NaCl droplet and **(B)** dyed 3.5% NaCl droplet.

Microscopic images of a NaCl droplet placed on the QCM electrode surface were observed with a confocal laser scanning microscope. Images of initial microdroplets formed around the primary droplet are shown in Figure 2A. The images reveal that a large number of microdroplets are formed around the primary droplet, and the size of the droplet decreases with the increase in distance from the primary droplet. Some microdroplets located close to the primary droplet begin to merge into a relatively bigger droplet. Although the primary droplet was dyed red in Figure 2B, the surrounding microdroplets remain colorless when the microdroplets just appeared. The color contrast between the primary droplet and the microdroplets can reflect the relationship between them. The results show that the microdroplets are not separated and migrated directly from the primary droplet; otherwise, the microdroplets will appear red consistent with the primary droplet.

### Mass Change of the Microdroplets

**FIGURE 3:**
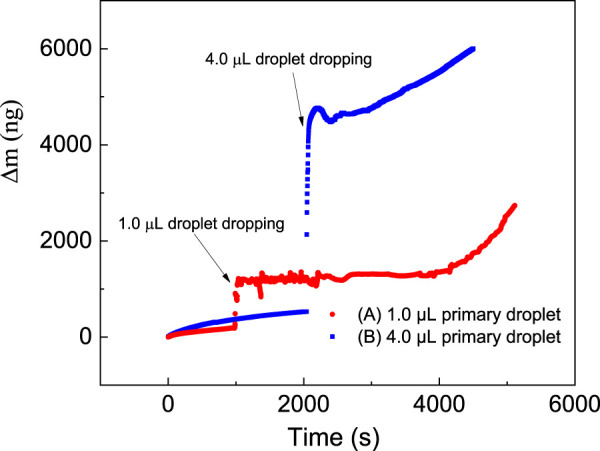
Change in the mass of an iron plated layer measured by QCM upon placing a NaCl droplet with a volume of **(A)** 1.0 μL and **(B)** 4.0 μL.

**FIGURE 4:**
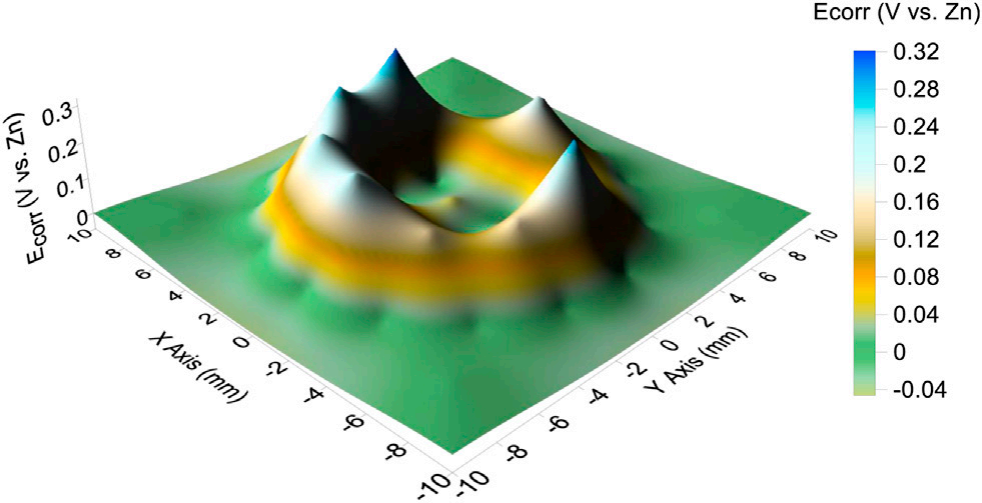
Corrosion potential distribution in the presence of a 3.5% NaCl droplet with a volume of 110 μL.

Figure 3 shows changes in the mass of the iron layer upon placing the NaCl droplet with a volume of 1.0 μL (Figure 3A) and 4 μL (Figure 3B), as measured by QCM.

For a primary droplet with a smaller volume of 1.0 μL, the mass slowly increases at first, after which it fluctuates following a dramatic increase, and finally increases rapidly. Before placing the NaCl droplet, the mass increases by a mere 212 ng in 1,000 s with an average rate of 0.212 ng/s. This slight increase in mass could be due to surface oxidation and moisture absorption. When the primary droplet is added at 1,000s, the mass change of the system immediately increases dramatically from 211 to 1,208 ng, reflecting the mass of the primary droplet. Simultaneously, the microdroplets formation is immediately observed. From 1,000 to 3,920 s, the mass variation curve for the microdroplet system shows fluctuating characteristics, sometimes increasing and decreasing at other times. However, the overall change in mass is relatively stable, only increasing from 1,134 to 1,302 ng. This fluctuation in the mass change curve indicates that factors that cause growth and decrease coexist in the system. From 3,920 s, the mass increases at a rapid rate of about 0.716 ng/s to reach 2,733 ng at 5,933 s.

For a primary droplet with a relatively larger volume of 4.0 μL, the mass change curve (Figure 3B) has a similar trend. The mass on the QCM crystal increased slightly to 527 ng in 2000 s before the initial drop was added. When a 4.0 μL saline droplet was added dropwise, the mass instantly increased to 4,564 ng. Subsequently, the mass on QCM began to grow rapidly after a period of stabilization. During the stabilization period from 2,103 s to 2,946 s, the mass only increased from 4,564 to 4,759 ng, although there was a relative mass maximum of 4,766 ng and a relative minimum of 4,493 ng. During the fast growth period, the average increase in mass is 0.796 ng/s.

### Corrosion Potential Distribution Under a Droplet

The corrosion potential distributions of the CTEA under a 110 μL droplet are mapped in Figure 4. The corrosion potential distribution on the electrodes exhibited a regular volcanic feature; that is, the edge of the droplet had a ring-shaped potential peak and the center of the droplet had a potential valley. The maximum corrosion potential under the droplet edge was 0.32 V, and the minimum corrosion potential under the center of the droplet was −0.04 V.

### Galvanic Current Distribution Under a Droplet

**FIGURE 5:**
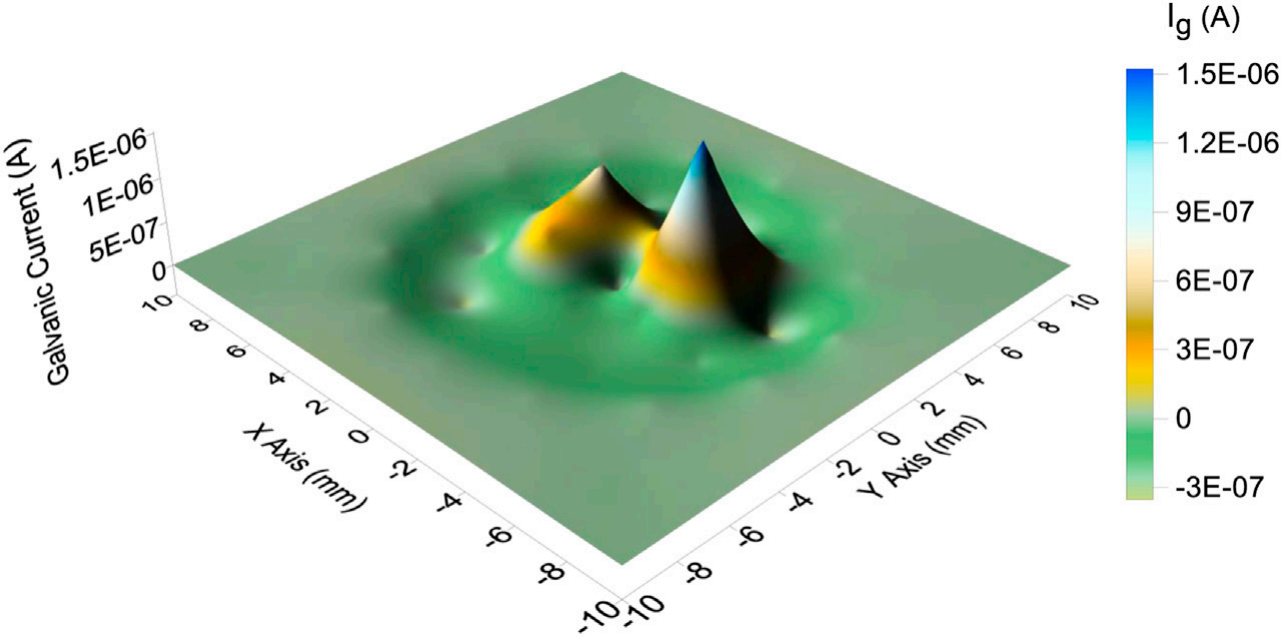
Galvanic current distribution in the presence of a 3.5% NaCl droplet with a volume of 110 μL.

The galvanic current distribution of the CTEA under the droplet with a volume of 110 μL is plotted in Figure 5. The result showed that several anodic galvanic current peaks existed in the central part of the droplet surrounded by a shallow cathodic galvanic current valley. Moreover, the maximum anodic galvanic current of the central part of the droplet was 1.5 μA. The cathodic current was relatively average and the minimum value was −0.3 μA.

### Macroscopic Observation of the Microdroplets

**FIGURE 6:**
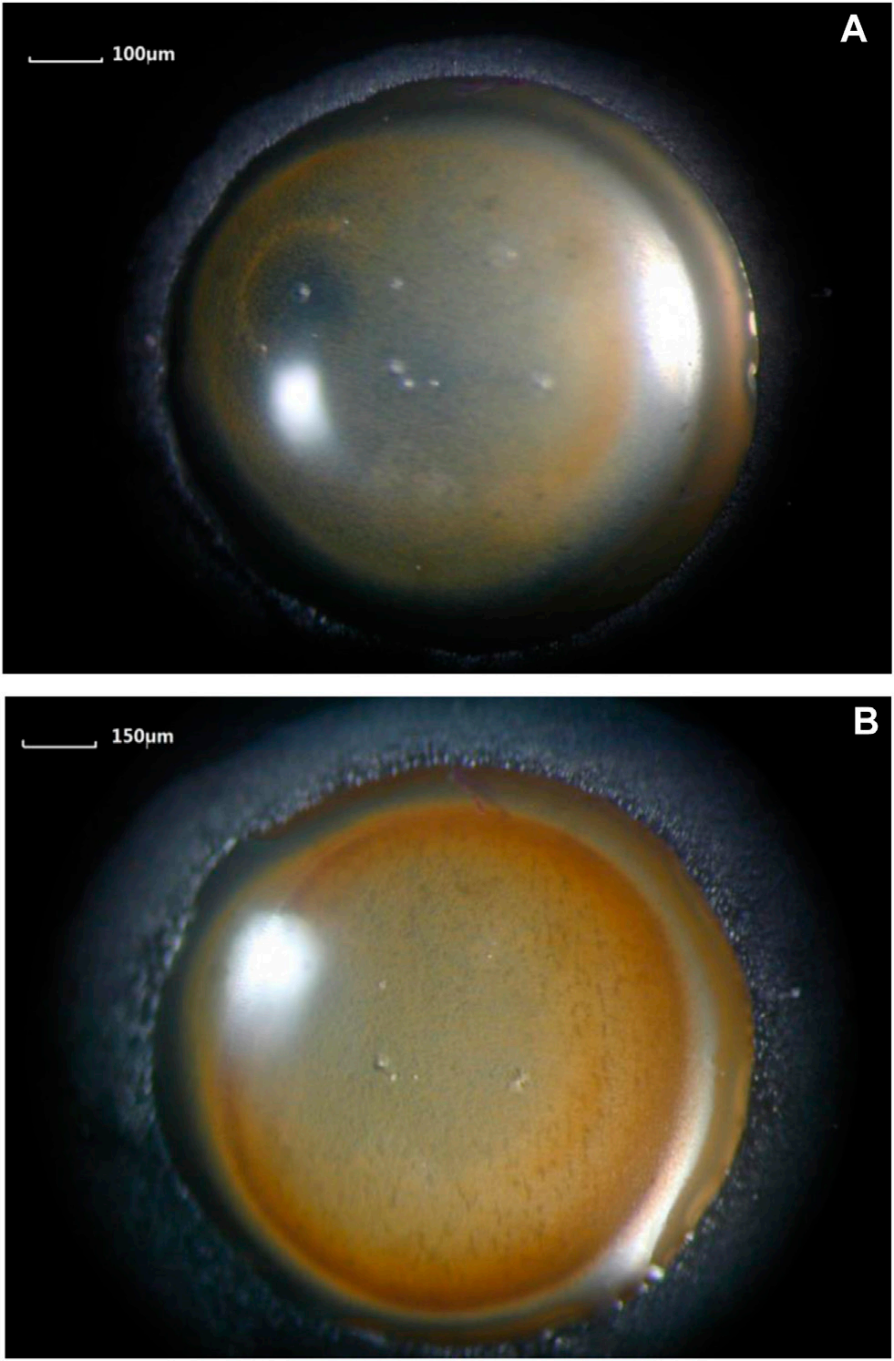
Morphological observation of the primary droplet and microdroplets system at different elapsed times after dropping the primary droplet with an initial volume of 1.0 μL.

The macroimages of the microdroplets system on the surface of the pure iron electrode were observed by a stereo microscope. Images taken 5 and 90 min after the dropwise addition of the saline droplet were selected to compare the changes in the morphology of the microdroplets system, as shown in Figure 6.

Compared with after elapsed time of 5 min, image of after elapsed time of 90 min showed that the primary droplet spreading area increased significantly, so did the microdroplets domain area, and the number of corrosion products increased significantly.

### Chemical Composition Measurement of the Microdroplets

**FIGURE 7:**
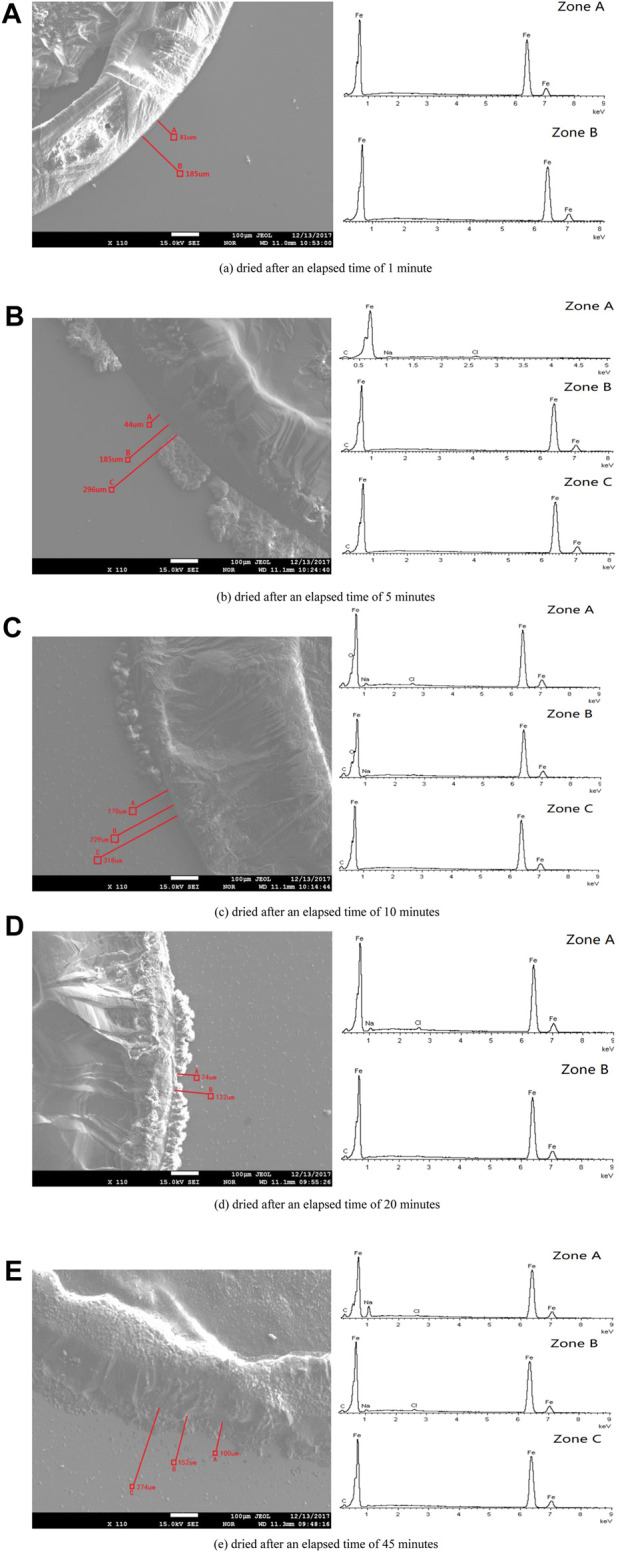
SEM images of the dried specimen’s surface and EDS results of the specific region marked on the left hand figure for different elapsed times after dropping the primary droplet. **(A)** Dried after an elapsed time of 1 min. **(B)** Dried after an elapsed time of 5 min. **(C)** Dried after an elapsed time of 10 min. **(D)** Dried after an elapsed time of 20 min. **(E)** Dried after an elapsed time of 45 min.

SEM and EDS were used to test the microchemical composition of different positions in the microdroplet domain region upon the pure iron electrode. Figure 7 shows the results of SEM/EDS analysis of the dried microdroplets zone for each specimen after elapsed times of 1, 5, 10, 20, and 45 min. Correspondingly, the EDS chemical composition measurements for all test zones are listed in Table 1.

**TABLE 1:** Chemical composition of different microdroplet domain zones by EDS, atomic number %.

Sample	d (μm)	C	O	Na	Cl	Fe
1 min (Zone A)	81					100
1 min (Zone B)	185					100
5 min (Zone A)	44			2.14	0.72	97.14
5 min (Zone B)	185	9.96				90.04
5 min (Zone C)	296	10.30				89.70
10 min (Zone A)	170		10.91	2.83	0.81	85.44
10 min (Zone B)	229	13.82	14.27	0.98		70.93
10 min (Zone C)	328	15.47				84.53
20 min (Zone A)	74			3.06	1.27	95.67
20 min (Zone B)	122	10.64				89.46
45 min (Zone A)	100	13.02		10.15	0.42	76.4
45 min (Zone B)	152	9.34		2.49	0.75	87.42
45 min (Zone C)	274	11.26				88.74

The above results show that the specimen in which the primary droplet remained for 1 min showed a clear droplet phase boundary edge after dehydration. EDS analysis of the zones 81 and 185 μm from the edge showed that only iron and no other chemical components was detected, indicating that the composition of the microdroplets in the initial stage was pure water and contained no salt.

When the primary droplet remained for a longer period of time, traces of precipitated salts appeared at the outer edges of the partial phase boundaries. This indicates that the outer boundary of part of the primary droplet merged with the adjacent microdroplet domain area, such that the boundary of the primary droplet began to expand outward.

Avoiding the area where the primary droplet expanded, several microdroplets domain zones at different distances from the initial primary droplet boundary were selected for chemical composition test. Results showed that some elements such as sodium, chlorine, oxygen, and carbon appear in some test results. If the interference element carbon and the basic element iron in the test results are neglected, other elements such as sodium, chlorine, and oxygen are inevitably related to the migration of the electrolyte and corrosion process.

Moreover, the positions containing sodium and chlorine elements were located close to the primary droplet boundary and appeared together. However, there were no sodium and chlorine elements at the distant positions. Among them, the special test results appeared in the sample retained for 10 min. Oxygen was detected in the nearer and farther positions, but not in all other samples. Moreover, only a small amount of sodium was detected in the farther position of this sample, and no chlorine appeared.

## Discussion

### Origin of Microdroplets

**FIGURE 8:**
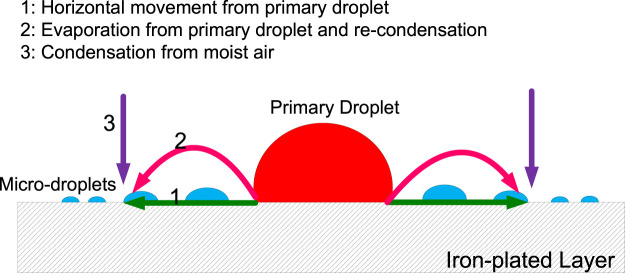
Schematic representation of possible microdroplet formation pathways.

In a sealed environment with an RH of 85%, microdroplets are formed on an iron-plated QCM electrode in the presence of a NaCl droplet. There are three possible pathways for the formation of microdroplets (Figure 8):

(1) Horizontal movement from the primary droplet along the metal surface (Tsuru et al., 2004);
(2) Evaporation from the primary droplet and recondensation on the metal surface (Liang et al., 2006);
(3) Condensation of moisture from the air

The microdroplets in pathways 1 and 2 are formed from water molecules in the primary droplet, with the water molecules migrating in a different manner. On the other hand, in pathway 3, the microdroplet formed is unrelated to the primary droplet but directly related to the water molecules in the gas phase environment.

Firstly, it is important to determine whether the microdroplet formation is related to the primary droplet. If the microdroplet formation occurs only via pathway 3, microdroplets could appear on the iron film even in the absence of the NaCl droplet in a sealed environment with 85% RH. However, we find that no microdroplets are formed before the primary droplet is placed on the iron film. Furthermore, the variation in mass during the first stage in Figure 3 is relatively stable. In other words, the experimental results prove that the microdroplets do not originate from the moist air alone, at least during the initial stages.

Therefore, from the above analysis, it could be concluded that the microdroplets originate from the water molecules present in the primary droplets during the initial stages. However, the manner of transfer, that is, whether the water molecules in the primary droplet migrate along the metal surface or through the gas phase for readsorption and condensation on the metal surface, needs to be further investigated.

In order to precisely determine the origin of the microdroplets, the primary NaCl droplet was dyed with red ink. If the microdroplets migrate from the primary droplet along the surface of the metal, then a continuous liquid film should exist in the zone connecting the edge of the primary droplet and microdroplets. If this is true, the microdroplets should also be tinged red. However, the experimental results show that the surrounding microdroplets are colorless in the initial stage, as observed in Figure 2B. Thus, pathway 1 could be excluded in the initial stage of microdroplets formation. This inference can also be confirmed from the chemical composition test results at the initial stage of the appearance of microdroplets. EDS analysis in Figure 7A showed that there were no other chemical components except iron existing in the initial microdroplets domain zone, indicating that the composition of the microdroplets in the initial stage was pure water and contained no salt. If pathway 1 is present immediately after the addition of the primary droplet, it is not possible to contain only water and no sodium chloride in the composition of the microdroplets. Therefore, it may be concluded that microdroplets are formed in the initial stage via pathway 2, that is, evaporation of water vapor from the primary droplet and recondensation on the metal surface.

As the microdroplets in pathway 1 come from the evaporated water in the primary droplets, this process does not affect the overall mass change of the microdroplets system. In the next developing stage of microdroplets, since the overall quality of the microdroplets system on QCM shows an increasing trend (Figure 3), it is necessary to clarify the source of microdroplets in this stage.

Among all three possible pathways, only pathway 3 could cause an increase in the mass of the microdroplets system because the microdroplets produced by this route are derived from the air outside the system. Therefore, in the developing stage of the microdroplets, there must be pathway 3; that is, the microdroplets system is capable of absorbing moisture from the external gas phase environment such that the microdroplets are enlarged and newly generated.

Based on analysis from the chemical composition, if microdroplets contain sodium chloride components, it is certainly obtained through pathway 1. In the system of microdroplets, only the primary droplet contains sodium chloride and the moist air is composed of pure gas phase. In general, it is difficult for the sodium chloride component to reach the microdroplets through pathway 2; that is, the transfer of the salt cannot be achieved by the recondensation process after evaporation. Then, the unique way to cause sodium chloride to be contained in the microdroplets is pathway 1; that is, the salt migrates through the invisible thin liquid film between the primary droplet and the microdroplets on the surface of the pure iron electrode.

When the sodium chloride droplet was dropped on the pure iron electrode for more than 5 min (Figure 7 and Table 1), sodium and chlorine elements were detected in the portion of the microdroplets covering area near the primary droplet boundary for all the samples. The measurement results demonstrate the contribution of pathway 1 to the microdroplets formation during the development of the microdroplets, but this pathway only affects the microdroplets domain region that is closer to the primary droplet.

In summary, in the initial stage of microdroplets formation, the only origin is the water derived from the edge of the primary droplet heat evaporating and recondensing at the surface of the electrode near the gas/liquid/solid three-phase boundary. In the developing stage of the microdroplets, the source contains three parts, that is, 1) horizontal movement from the primary droplet along the metal surface; 2) evaporation from the primary droplet and recondensation on the metal surface; and 3) condensation of moisture from the air.

### Formation Mechanism of Microdroplets

Previous research has shown that the microdroplets only appear when the primary droplet is added dropwise (Liang et al., 2006). When a NaCl droplet is dropped onto the QCM surface, the reactions that occur at the droplet/iron film interface could be

$$\text{Anodic}\;\text{reaction}\;\text{in}\;\text{the}\;\text{central}\;\text{region}:\text{Fe}\to {\text{Fe}}^{2+}+2e^{-},$$ (1)

$$\text{Cathodic}\;\text{reaction}\;\text{in}\;\text{the}\;\text{peripheral}\;\text{regions}:{\text{O}}_{2}+{2\text{H}}_{2}\text{O}+4e^{-}\to 4{\text{OH}}^{-}.$$ (2)

Fe^2+^ could be combined with OH^−^ and oxygenated into brown Fe(OH)_3_ during its diffusion into the fringe area. During the corrosion process under the primary droplet, only the oxygen dissolved into the electrolyte from air participates in the cathodic reaction and the subsequent oxidation of Fe^2+^. Thus, the entry of oxygen can result in an increase in the mass of the QCM electrode. Moreover, the generation of microdroplets around the primary droplet can also cause an increase in the overall mass of the system. However, the fluctuations in mass change imply that a process leading to mass reduction exists. Therefore, it is reasonable to deduce that the evaporation of the droplet significantly affects the total mass on the QCM surface.

We believe that the main reason for the evaporation of the primary droplet is the formation of a corrosion cell on the iron-plated layer under the droplet. In a 3.5% NaCl droplet/pure iron interface, the electrochemical potential difference between the anode and cathode was measured to be 0.36 V and acted as the driving force for the formation of microdroplets. Owing to the potential difference, a corrosion current circuit is formed between the anode and cathode. According to local galvanic current density measurement by CTEA (Figure 5), the maximums of anodic and cathodic electric current density of pure iron under the NaCl droplet are 764 and −152 μA/cm^2^, respectively. The motion of charges due to the presence of the electric field gives rise to an electric current that causes the temperature in the liquid film to increase owing to the Joule heating effect (Sharma et al., 2013). Electrochemical reaction in corrosion cell can also generate heat which will help evaporation of the primary droplet. For the electrochemical parameters obtained from Figure 4 and Figure 5, the maximum potential difference between anode and cathode is 0.36 V, and total anodic current is 3 μA; then, the Joule heat in an hour could be obtained by the formula Q = UIt. The estimated Joule heat is 3.6 mJ for the 3.5% NaCl droplet with a volume of 110 μL. The generated Joule heat helps accelerate the evaporation process of primary droplet. Furthermore, the cathodic polarization of the three-phase line reduces the surface tension of the interface between the primary droplet and the metal (Wang et al., 2008). In this condition, the local water vapor around that area becomes supersaturated. Therefore, the corrosion cell could accelerate water evaporation by the combined action of cathodic polarization and the thermal effect composed of Joule heat and corrosion reaction heat. Considering the film thickness and strength of the electric field, one can expect a relatively higher rate of evaporation at the three-phase boundary. Consequently, the humidity of the air near the three-phase boundary is liable to reach the supersaturated state first, resulting in the recondensation of water vapor that evaporates from the primary droplet. Thus, evaporation from the primary droplet and recondensation on the surface near the three-phase boundary can be regarded as the main origin of the microdroplets in the initial stages. This type of mechanism is also indicated by the distribution of microdroplets in Figure 1; that is, the size of the microdroplets decreases in the direction away from the primary droplet.

Based on the above analysis, the fluctuation in the initial mass change of the iron film after droplet addition in Figure 3 is attributed to the interactions among droplet evaporation, oxygen absorption reactions, and moisture adsorption and condensation on the surface. The influence of droplet evaporation, corrosion reaction, and vapor condensation on the mass change of the quartz crystal is almost in a dynamic equilibrium state.

With the increase in elapsed time, the surface mass of the QCM electrode begins to increase significantly. During this period, the number of microdroplets covering the area increases significantly, and the microdroplets near the primary droplet begin to merge, enlarge, and partially connect with the primary droplet. The increase in the total mass of the system as well as the size and coverage area of the microdroplets indicates that condensation from moist air, that is, pathway 3, becomes the main source of microdroplets in the developing stage.

**FIGURE 9:**
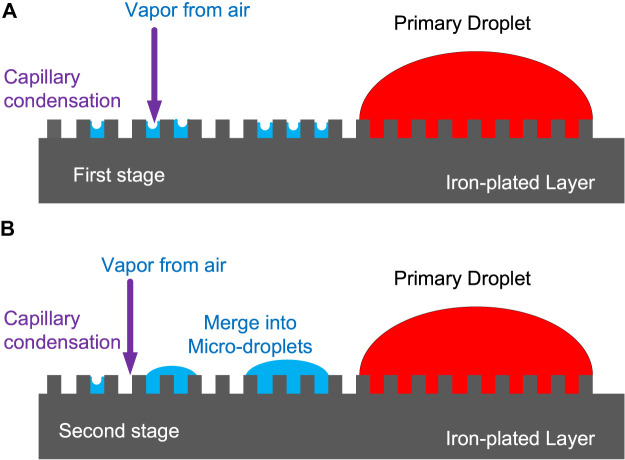
Schematic diagram of nucleation and growth of microdroplets.

In order to clarify the nucleation and growth mechanism of microdroplets from the gas phase on the surface of the metal electrode, we have drawn a schematic diagram (Figure 9) to describe the transition process of microdroplets from the initial stage to the developing stage. In the initial stage, there is a relatively higher concentration of water vapor in the vicinity of the three-phase boundary after the evaporation of the primary droplet. Thus, the water vapor in the air first nucleates and develops around the primary droplets. If the moisture comes from the vapor in the gas phase, it can condense even on the surface of a typical hydrophobic lotus leaf (Cheng and Daniel, 2005). In fact, the electroplated iron film layer on QCM is not a completely flat surface, and the microstructure exhibits a rugged state. A small gap is created between adjacent protrusions. The water vapor molecules both in the air and from evaporation of primary droplet tend to be trapped in these gaps in the first stage, as shown in Figure 9A, thereby forming a concave liquid surface, reducing the saturated vapor pressure, and facilitating the condensation of water vapor due to the capillary condensation effect.

In the second stage, adjacent tiny condensed droplets will merge into microdroplets, as shown in Figure 9B. The microdroplets initially formed around the primary droplet can significantly alter the microscopic geometry of the surface of the QCM electrode. As a result, water vapor in the air tends to reach a localized supersaturation state owing to capillary condensation in the microdroplet domain region, resulting in the aggregation of existing microdroplets and generation of new microdroplets.

As discussed in the section Origin of Microdroplets, there also exists the horizontal movement along the metal surface of the electrolyte during the developing stage of microdroplets. The negatively charged three-phase boundary caused a decrease in local surface tension of liquid/metal interface (Wang et al., 2008). The surrounding area of the primary droplet became more hydrophilic due to the joint effect of cathodic polarization induced decrease of surface tension and capillary condensation. The large density and size of the microdroplets near the edge of the primary droplet caused a local increase in the concentration of vapor in the surrounding air; thus, the metal surface between the primary droplet and the microdroplet in this region can easily adsorb water vapor to form a thin layer. The thin liquid film can connect the primary droplet and the microdroplets. Thereby a passage for the electrolyte to migrate the microdroplets from the main droplets is produced. Therefore, the presence of a thin liquid film channel is a prerequisite for the occurrence of pathway 1 of the microdroplets source. From the test results shown in Table 1, the chemical components that migrated through pathway 1 to the surrounding microdroplets can be analyzed. It can be found that the ratio of sodium element to chlorine element measured in the microdroplets coverage area near the primary droplet boundary is greater than 1. Typically, only sodium was detected in the 10 min (Zone B) sample, and no chlorine was found. The reason accounting for this result is that the connection of the primary droplet with nearby microdroplets causes them to form a typical oxygen concentration corrosion cell, which accumulates charge and rapidly absorbs oxygen in the air to occur an oxygen reduction reaction (2), producing OH^−^. Thus, Na^+^ in the primary droplet migrates through the thin liquid film to balance with OH^−^ existing in the microdroplets to achieve electrical neutrality. Therefore, the microdroplets around the primary droplet are alkaline due to the edge cathodic reaction and contain sodium ions. This is consistent with the results in the literature (Tsuru et al., 2004). The above explanation can also be confirmed by the presence of oxygen measured in both 10 min (Zone A) and 10 min (Zone B) samples.

**FIGURE 10:**
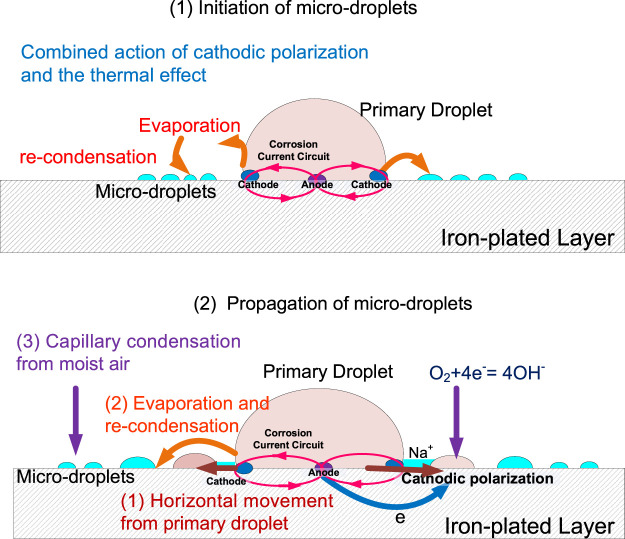
Schematic representation of microdroplets formation mechanism.

Based on the above discussion, we present a model to illustrate the formation mechanism of microdroplets; the schematic diagram is shown in Figure 10.

First, in the initial stage of microdroplets, the water molecules evaporate from the primary droplet due to the combined action of cathodic polarization and the thermal effect composed of Joule heat and corrosion reaction heat, which results from the corrosion cell under the primary droplet. The evaporated water molecules recondense in the vicinity of the three-phase boundary to form microdroplets, as shown in Figure 10 (1). The microdroplets initially formed around the primary droplet can significantly alter the geometry of the metal surface at the microscopic level. As a result, the water vapor in the air tends to reach a localized supersaturation state owing to capillary condensation in the microdroplet domain region, resulting in the aggregation of existing microdroplets and generation of new ones. An oxygen concentration cell is generated by a thin liquid film connection between the primary droplet and the nearby microdroplets, wherein the microdroplets are subjected to cathodic polarization to occur an oxygen reduction reaction. The Na^+^-containing electrolyte in the primary droplet is driven to migrate horizontally along the electrode surface to the microdroplets. Therefore, as shown in Figure 10 (2), the propagation of microdroplets in the developing stage attributes to horizontal movement of the electrolyte, water evaporation, and recondensation from primary and capillary condensation from moist air.

### Implication to Marine Atmospheric Corrosion

Observation of microdroplets system on the surface of the pure iron electrode (Figure 6) implied the correlation between microdroplets and atmospheric corrosion. After 90 min, the coverage area of the primary droplet and the microdroplets increased significantly. Obviously, the microdroplets system can expand around the primary droplet upon the pure iron surface in an 85% RH atmospheric environment. At the same time, the primary droplet also gradually expanded its volume and coverage by incorporating the surrounding microdroplets. Due to the difference in oxygen concentration to form the differentiation of the anode and cathode, the interfacial electrochemical reaction occurred, and the microdroplets act as a cathodic reaction zone to produce OH^−^. Some microdroplets located close to the primary droplet began to merge into a relatively bigger droplet, as shown in Figure 2. During the developing stage, the number of microdroplets covering the area increased significantly and the microdroplets near the primary droplet began to merge, enlarge, and partially connect with the primary droplet. When the moisture in the air was further agglomerated, a water molecular membrane is formed between the primary droplet and the neighboring microdroplets. At this time, Na^+^ in the primary droplet could diffuse into the adjacent microdroplets of microdroplets through this layer of water molecules. This process can be seen as the previous step in the expansion of the primary droplet. Thus, the formation of microdroplets can promote the spreading of saline droplets upon the metal surface.

On the other hand, the formation of microdroplets can cause an increase in the volume of the primary droplet, which in turn affects the development of the microdroplets. Comparing the difference between the mass change curves of the primary droplets with different volumes on the surface of the iron-plated layer in Figure 3, it can be found that the duration of the mass stabilization phase for 4.0 μL droplet is significantly shorter than that of 1.0 μL, from 2,920 s to 843 s. The mass change of 1 μL after 4000 s is significantly high and abrupt for microdroplets formation or partial condensation from the air. Compared with the main droplet of 4.0 μL, the stable period of the mass change curve of the 1 μL droplet system is relatively longer, and the growth rate in the later period is faster. The possible reason is that the volume of the microdroplets produced by the small-volume main droplet is relatively small, and its size and geometry are more sensitive to the condensation of moisture in the air in this area. Meanwhile, the presence of microdroplets could accelerate the merger of thin electrolyte layers distributed at various locations to form a continuous thin film, which is a necessary condition for atmospheric corrosion to take place.

Therefore, the generation of microdroplets can undoubtedly accelerate and promote marine atmospheric corrosion.

## Conclusion

During the initial stage, the microdroplets mainly originate from the recondensation of water vapor in the vicinity of the three-phase boundary after the evaporation of the primary droplet due to the combined action of cathodic polarization and the thermal effect, which result from the corrosion cell under the primary droplet. The maximal electrochemical potential difference between the anode and cathode was measured to be 0.36 V and acted as the driving force for the formation of microdroplets. The maximums of anodic and cathodic electric current density of pure iron under the NaCl droplet are 764 and −152 μA/cm^2^, respectively.

In the developing stage, the microdroplets mainly originate from the condensation of moisture in the air derived from the capillary condensation effect. Propagation of microdroplets in the developing stage attributes to horizontal movement of the electrolyte, water evaporation, and recondensation from primary and capillary condensation from moist air.

The initiation and propagation of microdroplets can promote and accelerate marine atmospheric corrosion.

## Data Availability

The original contributions presented in the study are included in the article/Supplementary Material; further inquiries can be directed to the corresponding author.
